# Health Hazards Associated with Exposure to Endosulfan: A Mini-Review

**DOI:** 10.3390/toxics13060455

**Published:** 2025-05-29

**Authors:** Agnieszka Berdowska, Katarzyna Bandurska

**Affiliations:** Department of Dietetics and Food Studies, Faculty of Science and Technology, Jan Dlugosz University, 42-200 Czestochowa, Poland; k.bandurska@ujd.edu.pl

**Keywords:** endosulfan, organochlorine pesticide, human health, neurotoxicity, persistent organic pollutants, acute poisoning

## Abstract

Endosulfan, a persistent organochlorine pesticide, has raised global concern due to its toxicological effects on human health and the environment. The popularity of endosulfan was driven by its effectiveness and low cost compared to alternative insecticides. The compound’s environmental persistence and bioaccumulative properties also contributed to its sustained use over several decades. Despite regulatory bans in many countries, residues of endosulfan continue to be detected in soil, water, and food sources, posing a threat through chronic exposure. Although endosulfan has been listed in the Stockholm Convention as a persistent organic pollutant targeted for global elimination, it is still used illegally in some countries. This mini-review synthesizes current knowledge on its toxicological profile, including neurotoxicity, endocrine disruption, reproductive toxicity, potential carcinogenicity, and acute poisoning, based on the latest scientific literature. The paper also highlights current regulatory frameworks, historical usage trends, global distribution and alternatives to endosulfan in agriculture. Understanding the scope of its health impacts and ongoing risks is crucial for policymakers, researchers, and public health authorities seeking to protect populations from legacy pollutants. In addition, recognizing the long-term impacts of endosulfan is essential for effective health risk assessment, environmental monitoring, and the promotion of safer alternatives.

## 1. Introduction

Endosulfan is a cyclic sulphurous acid ester with a molecular weight of 407 Da (Daltons), belonging to the class of cyclodienes [1]. It is an organochlorine insecticide used to protect crops such as fruits, vegetables, cereals, oilseed crops, coffee, tea, tobacco, and cotton plantations. It is also used for pest control in home gardens [2,3,4] and as a wood preservative [5]. Endosulfan, despite its toxic effects, is still one of the pesticides commonly used in agriculture, particularly in developing countries [1,6,7,8]. This is due to its effectiveness, low cost, and environmental stability [9].

Endosulfan residues contaminating the environment bioaccumulate and biomagnify in the food chain, causing adverse effects on non-target organisms, including humans [8].

Endosulfan has been banned in many countries. However, it is important to note that it can still be used illegally [10]. For example, Sharma et al. [11] showed the presence of endosulfan in vegetables from the north-western Himalayan region of India collected in 2021, although endosulfan has been banned in India since 2011 [12]. Additionally, it is quite persistent and can be detected in the environment even after many years. Ben Mukiibi et al. [13] showed the presence of endosulfan in soil samples collected in 2020 from a shallow depth (<20 cm) in the vicinity of an abandoned pesticide store in western Uganda that operated in the 1960s and 1970s.

Endosulfan has a toxic effect on various human systems, such as the central nervous system (CNS), the cardiovascular, immune, and reproductive systems [1,14]. It is also genotoxic and disrupts hormonal balance [15]. There are reports in the literature that endosulfan delays puberty in boys [16], increases the risk of developing neural tube defects, anencephaly, and spina bifida [17]. It has also been reported to contribute to preterm birth [18], although there are also reports of no such association [19]. Endosulfan can cause rhabdomyolysis, which then leads to renal tubular damage, resulting in acute tubular necrosis and ultimately renal failure [20]. Endosulfan has been linked to the development of cancers, such as breast cancer [21,22,23], and is also associated with a worse prognosis in patients with various types of cancer (breast, thyroid, prostate, skin, digestive system, uterus and ovaries, lung, liver and blood) [24]. Acute endosulfan poisoning in humans causes symptoms of nervous system excitation, resulting in nausea, vomiting, headache, dizziness, paresthesias, focal myoclonic seizures, and generalized seizures [1,25,26]. Later complications of acute endosulfan poisoning include the following: rhabdomyolysis, hepatic toxicity, hypotension, acute kidney injury, and thrombocytopenia. Acute endosulfan poisoning can result in death in humans, most often caused by refractory status epilepticus, multiple organ failure, or acute renal failure with metabolic acidosis [25].

New studies indicate that endosulfan may be associated with human cancer. A recent report showed that in vitro, endosulfan promotes cell growth, migration, and invasion in human breast adenocarcinoma MCF-7 cells [27]. Moreover, the increasing number of reports on the relationship between the presence of endosulfan in human blood or tissues and the development of cancer is alarming [21,22,24,28].

The article synthesizes current knowledge on the impacts of endosulfan on human health and highlights current regulatory frameworks, historical usage trends, global distribution, and alternatives to endosulfan in agriculture.

## 2. Structure and Chemical Properties of Endosulfan

Endosulfan (6,7,8,9,10,10-hexachloro-1,5,5a,6,9,9a-hexa-hydro-6,9-methano-2,4,3-benzodioxa-thiepin-3-oxide) is an organochlorine insecticide with the chemical formula C₉H₆Cl₆O₃S. This compound is toxic, bioaccumulative, and persistent in the environment. It has been marketed under various trade names, including Cyclodan, Endosol, Insectophene, Thiodan, and Thiosulfan, among others. Structurally, it belongs to the cyclodiene group and contains a sulfite ester group, contributing to its environmental persistence. In particular, the presence of sulfite ester groups contributes to the molecule’s resistance to hydrolysis under both acidic and basic conditions. Endosulfan exists in two stereoisomeric forms: α-endosulfan (also called endosulfan I) and β-endosulfan (endosulfan II), typically found in a 7:3 ratio in commercial formulations. These isomers differ in their spatial configuration, affecting their physicochemical properties and toxicological behavior [2,29,30,31,32].

The main degradation product of endosulfan is endosulfan sulfate, which is also a toxic and persistent metabolite with similar properties to the non-degraded compound [33]. Physically, endosulfan appears as brown-colored solid crystals and has a slight sulfur dioxide odor. Endosulfan remains in soils as a result of its extensive historical use, with a half-life of ~60 days for α-endosulfan and up to 800 days for β-endosulfan [33,34]. Endosulfan can be released into the atmosphere through spray drift, post-application volatilization, and wind erosion of soil particles [33].

The majority of organochlorine insecticides that were widely used in agriculture are now banned in most countries, yet their residues persist in soil [35].

Endosulfan has been used on a variety of crops, including broccoli, potatoes, coffee, cotton, peaches, apples, nectarines, prunes, lettuce, tomatoes, grapes, melons, cauliflower, carrots, cabbage, rapeseed, strawberries, alfalfa, beans, cereals, cucumbers, tobacco, tea, oil crops, as well as ornamental flowers for pest control [36].

## 3. History, Global Distribution, and Regulatory Control of Endosulfan Use

Endosulfan was developed in the early 1950s by the German company Hoechst AG and introduced to the U.S. market in 1954. Due to its broad-spectrum efficacy against various insect pests, it gained widespread adoption in agriculture, particularly in cotton, tea, coffee, rice, and vegetable cultivation [29].

From 1958 to 2000, global production of endosulfan was estimated at approximately 18,000–20,000 tonnes annually, resulting in a cumulative usage of around 300,000 tonnes. India was the largest consumer, accounting for about 113,000 tonnes during this period, followed by the United States with 26,000 tonnes. China’s annual usage averaged around 2,800 tonnes between 1998 and 2004. The compound was also extensively used in countries including Brazil, Australia, and several Southeast Asian and African nations [29,30,37].

In May 2011, during the fifth meeting of the Conference of the Parties (COP5) to the Stockholm Convention on Persistent Organic Pollutants (POPs), representatives from 127 governments agreed to list endosulfan in Annex A of the Convention. This listing mandates the elimination of endosulfan production and use worldwide, with specific exemptions for certain agricultural applications. The decision aimed to phase out endosulfan by 2012, acknowledging its detrimental effects on human health and the environment [38].

In response to the Stockholm Convention’s amendment, the European Union (EU) implemented measures to prohibit the production, marketing, and use of endosulfan. The legal act enforcing this ban came into effect in July 2012. Subsequently, the export of endosulfan was banned under Regulation (EU) No. 649/2012, effective from 1 April 2013. These actions align with the EU’s commitment to eliminating persistent organic pollutants in accordance with international agreements. Additionally, Regulation (EU) 2019/1021 on persistent organic pollutants lists endosulfan, reinforcing its prohibition within member states [39,40,41].

Moreover, the use of endosulfan worldwide is regulated by major international and national authorities, including the Environmental Protection Agency (EPA) and the World Health Organization (WHO) [42,43].

## 4. Toxicity of Endosulfan and Associated Health Hazards in Various Organisms

Toxicity refers to the inherent ability of a substance to cause adverse effects in living organisms. The degree of toxicity depends on several factors, including dose, duration, and route of exposure, the chemical’s bioavailability, and the organism’s susceptibility. A substance is considered toxic when, under specific conditions, it induces harmful effects such as cellular damage, disruption of physiological processes, or death. Toxic effects can be acute (resulting from a single or short-term exposure) or chronic (arising from long-term, low-level exposure).

Median lethal dose (LD_50_) values for endosulfan vary depending on species, sex, and nutritional status of the animal [44].

Silva et al. [45] conducted a comprehensive toxicological assessment of endosulfan, highlighting its moderate acute toxicity in rodent models. The oral LD₅₀ was found to be 48 mg/kg for male rats and as low as 10 mg/kg/day for females following gavage administration. In dermal exposure studies, the LD₅₀ was 130 mg/kg/day for males and 70 mg/kg/day for females. Inhalation exposure produced a lethal concentration 50% (LC₅₀) of 34.5 mg/L in males and 12.6 mg/L in females, again demonstrating greater susceptibility in females across all exposure routes. These results indicate that females are two- to five-times more sensitive to endosulfan toxicity than males, depending on the route of exposure. Gupta et al. [46] observed that mice appear to be quite sensitive to endosulfan’s lethal effects, with a reported LD_50_ value of 7.36 mg/kg in males. Critical No-Observed-Effect-Level (NOEL) values were also identified: 0.7 mg/kg/day for acute oral exposure (rabbit developmental studies), 1.2 mg/kg/day for subchronic dietary exposure (rat reproduction studies), and 0.6 mg/kg/day for chronic oral exposure. Inhalation NOELs were notably low, estimated at 0.001 mg/L (acute/subchronic) and 0.0001 mg/L (chronic). Although no evidence of carcinogenicity or genotoxicity was observed in rodents, neurotoxic effects were significant and correlated with the observed reproductive toxicity in males, particularly affecting sperm at neurotoxic doses. Endocrine-related effects, such as cytochrome P450 induction, were noted but were reversible. Importantly, developmental and reproductive toxicity occurred only at doses that also induced neurotoxicity, and fetuses or juveniles were not more sensitive than adults [45].

Kumar et al. [47] observed that acute exposure to endosulfan caused pronounced biochemical, immunological, and histopathological damage in *Chanos chanos* juveniles. This toxic response was closely linked to oxidative stress and the disruption of key enzymatic pathways, such as the inhibition of acetylcholinesterase (AChE) and the elevation of liver transaminases. The 96 h LC₅₀ value for pure endosulfan (α:β ratio of 7:3) under static non-renewable conditions was determined to be approximately 20.5 mg/L (tested range: 18.5–22.5 mg/L). Significant sublethal effects were observed, including: Increased activity of antioxidant enzymes (catalase (CAT), superoxide dismutase (SOD), glutathione-S-transferase (GST)) in the liver, gills, and brain in a dose- and time-dependent manner; marked inhibition of AChE activity in the brain, indicating neurotoxic effects; elevated activity of metabolic enzymes (lactate dehydrogenase, alanine aminotransferase (ALT), aspartate aminotransferase (AST), glucose-6-phosphate dehydrogenase) in the liver and gills; increased blood glucose and cortisol levels, suggesting physiological stress and decreased respiratory burst activity, indicating immune suppression. Histopathological alterations were observed in both the gills and liver of exposed fish. In the gills, changes included curling and fusion of the secondary lamellae, thickening of the primary epithelium, epithelial hyperplasia, the presence of aneurysms, and lamellar collapse. In the liver, moderate doses of endosulfan induced cloudy swelling, necrosis, and pyknotic nuclei, while higher doses (21.5 mg/L) led to severe hepatic necrosis.

An experiment conducted by the Kumari group [48] explored the chronic effects of endosulfan exposure on immune function in African catfish (*Clarias gariepinus*), revealing significant immunosuppression and increased vulnerability to microbial infections. Chronic exposure to endosulfan has been shown to exert immunotoxic effects in *C. gariepinus*, compromising the fish’s immune response and significantly increasing susceptibility to microbial infections. The 72 h LD_50_ of *Aeromonas hydrophila* for African catfish was determined to be 1 × 10⁸ CFU/mL, indicating a high susceptibility of this species to bacterial infection under chronic endosulfan exposure. Long-term contact with sublethal concentrations of this organochlorine pesticide resulted in impaired defense mechanisms, including reduced leukocyte activity and altered immune biomarkers.

The study by Capkin et al. [49] clearly demonstrates the high acute toxicity of endosulfan in juvenile rainbow trout (*Oncorhynchus mykiss*), with lethality increasing sharply over time. The low LC₅₀ values, beginning at 19.78 μg/L (24 h) and dropping to 1.75 μg/L (96 h), highlight the potent and time-dependent toxic effects of this pesticide. Histopathological alterations, including severe liver necrosis and irreversible gill damage, further confirm its systemic toxicity. Moreover, the findings underscore that environmental parameters—such as water temperature, alkalinity, and fish size—can significantly influence toxicity outcomes. Notably, survival rates were significantly influenced by fish size, with smaller fish showing higher sensitivity, and by environmental conditions such as temperature and water alkalinity. Increased alkalinity (≥42 mg/L as CaCO₃) notably improved survival, whereas total water hardness had no significant effect.

Nam et al. [50] conducted a study highlighting the acute toxicity of α-endosulfan and selected polycyclic aromatic hydrocarbons—specifically fluorene and phenanthrene—toward the earthworm *Eisenia fetida* under artificial soil conditions. The LC₅₀ values determined for *E. fetida* were 9.7 mg/kg for α-endosulfan, 133.2 mg/kg for fluorene, and 86.2 mg/kg for phenanthrene, demonstrating the substantially higher toxicity of α-endosulfan. When combined at LC₁₀ and LC₅₀ concentrations, α-endosulfan and fluorene exhibited a synergistic toxic effect, significantly increasing earthworm mortality. Additionally, biochemical responses, such as the upregulation of GST and HSP70 gene expression, confirmed enhanced cellular stress under co-exposure.

An analysis by Uğurlu et al. [51] investigated the acute toxicity and histopathological effects of commercial-grade endosulfan on the freshwater amphipod *Gammarus kischineffensis*. The 96 h LC₅₀ value was determined to be 1.861 mg/L, indicating that endosulfan is highly toxic to this aquatic invertebrate. Sublethal exposure resulted in significant histopathological damage to the gill tissues, including pillar cell hypertrophy, atrophy of the hemocoelic space, and hemocytic infiltration.

These results clearly demonstrate the potent toxic impact of endosulfan on various organisms and emphasize the need for stricter environmental regulations to mitigate its ecological risks and protect ecosystem health.

## 5. Routes of Entry into the Human Body and Sources of Exposure

Endosulfan can enter the human body in different ways. It can be ingested [52] (including through breast milk [53]), through the skin [54], by inhalation, and even transplacental transmission from mother to fetus is possible [1].

Endosulfan exposure may be either accidental or intentional. Sources of exposure include a contaminated environment, contaminated food, occupational exposure [55], but also deliberate ingestion for suicidal purposes [56]. Food sources of endosulfan may include not only fruits or vegetables directly contaminated with this insecticide or grown in contaminated soil, but also honey collected by bees in areas where endosulfan was previously used [13], river fish [10], and even seafood [57]. For example, Ben Mukiibi et al. [13] detected endosulfan in honey from beehives that were located at linear distances of 125 to 550 m from the pesticide store that had been closed for many years. The average amounts in honey were 10.8 µg/kg for α-endosulfan and 0.6 µg/kg for β-endosulfan. Arisekar et al. [10] detected endosulfan in 2020 in amounts ranging from 17.95 to 26.05 μg/kg in rohu fish (*Labeo rohita*) from the Thamirabarani River in India. Kido et al. [57] found this insecticide in shrimp bought at the fish markets in Japan between 2018 and 2020 and originating from Japanese coastal waters (ranging from 2.87 to 18.00 ng/g dry weight) and from the South China Sea (ranging from 3.47 to 5.54 ng/g dry weight). Endosulfan can also be found in the air, not only in agricultural areas, but also in urban areas [58], and it has even been found in the air of subarctic zones [59]. Smoking contaminated tobacco may also be a source of endosulfan exposure [4].

## 6. Endosulfan Tissue Distribution and Excretion in Humans

Endosulfan is highly lipophilic, which means it is rapidly absorbed and then distributed in tissues, including the central nervous system. The slow release of endosulfan from its lipophilic deposits back into the circulation prolongs its duration of action. The half-life of endosulfan is 1–7 days [1,3,60]. In individuals exposed to endosulfan, the compound was detected in the highest concentrations in adipose tissue [61], confirming its lipophilicity. Although endosulfan is deposited in adipose tissue, recently Fan et al. [62] demonstrated no relationship between serum endosulfan levels and body mass index (BMI). In exposed individuals, endosulfan was also found in the blood [55,61,63] (including cord blood [64]), urine [65], as well as in the milk of breastfeeding women [53] and the placenta of pregnant women [19].

Endosulfan metabolites are eliminated from the human body in urine and feces. Alpha and beta endosulfan isomers and the following metabolites were found in urine: diol, ether, lactone, and sulfate [66].

## 7. Mechanisms of Endosulfan Toxicity

**Figure 1:**
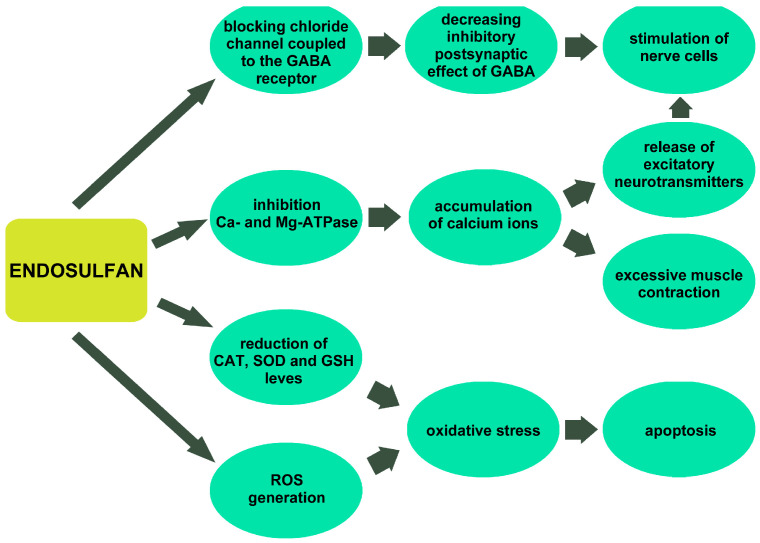
Mechanisms of endosulfan toxicity. CAT—catalase, GABA—γ-aminobutyric acid, GSH—glutathione, ROS—reactive oxygen species, SOD—superoxide dismutase.

Endosulfan is called a non-competitive γ-aminobutyric acid (GABA) antagonist [67]. It does not bind directly to the GABA binding site. Instead, it binds to the chloride channel coupled to the GABA receptor, blocking it. In this way, it inhibits GABA attachment to its receptor [1]. Physiologically, GABA binding to its receptors causes an influx of chloride ions into neurons, which in turn causes hyperpolarization of cell membranes and, as a result, reduces neuronal excitability. Endosulfan decreases the inhibitory postsynaptic effect of GABA at its receptor. This leads to uninhibited stimulation of nerve cells [1,68,69]. In addition, endosulfan can inhibit Ca- and Mg-ATPases, which leads to the accumulation of calcium ions and causes excessive release of excitatory neurotransmitters [1]. Moreover, endosulfan may cause oxidative stress by inducing reactive oxygen species (ROS) and membrane lipid peroxidation [70]. Endosulfan-induced oxidative stress activates caspase 3 and NFkβ, which may lead to apoptosis [14]. Endosulfan has been shown to significantly reduce glutathione content in a dose-dependent manner in human peripheral blood mononuclear cells (PBMC) in vitro, demonstrating a close correlation between oxidative stress and the degree of apoptosis [71,72]. Oxidative stress can cause irreversible cellular component modification, leading to damage or even cell death [2,73]. Figure 1 schematically illustrates mechanisms of endosulfan toxicity.

## 8. Endosulfan Neurotoxicity in Humans

The most important toxic effects of endosulfan in humans include neurotoxicity [74]. Endosulfan causes hyperstimulation of the central nervous system. Therefore, it lowers the seizure threshold. Typical symptoms of endosulfan poisoning in the central nervous system include nausea, vomiting, headache, agitation, paraesthesias, dizziness, lack of coordination, confusion, myoclonus, focal seizures, and generalized seizures [1,25,26]. Severe endosulfan poisoning can cause status epilepticus, which can be fatal due to asphyxia [26]. By affecting the frontal cortex, endosulfan causes cognitive impairment and behavioral deficits [75]. Visual disturbances have also been reported in cases of endosulfan poisoning. They were caused by damage to the occipital cerebral cortex, without accompanying dysfunction of the optic nerve or retina [76].

Endosulfan may be one of the causes of amyotrophic lateral sclerosis (ALS). ALS is a progressive degeneration of spinal and cortical motor neurons. The causes of this disease are unclear. Risk factors include family history and environmental pollution. Talbott et al. [77] found that the risk of ALS increased with every 10 ng/g of serum α-endosulfan.

## 9. Endosulfan Genotoxicity

Both α- and β-endosulfan are genotoxic. Lu et al. [78] studied in vitro the effect of endosulfan on the frequency of sister chromatid exchanges (SCE), micronuclei (MN), and DNA damage assessed by single-cell gel electrophoresis (SCG) using the human hepatoma cell line HepG2. While both induced DNA strand breaks, only β-endosulfan showed a significant effect in the SCE and MN tests on HepG2 cells. The damaging effect of endosulfan on HepG2 line cells was later confirmed in the study by Li et al. [79].

Sebastian and Raghavan [80] demonstrated in vitro that endosulfan induces ROS-mediated DNA damage in the human erythroleukemic cell line K562 and the human leukemic cell line Reh. Endosulfan induces ROS in a concentration- and time-dependent manner in cell lines. Furthermore, they found that this compound additionally has a detrimental effect on DNA repair by promoting misrepair, exacerbating deletions and mutations. Thus, endosulfan exposure may lead to genomic instability.

## 10. The Effect of Endosulfan on the Reproductive System and Fetal Development

Endosulfan has an adverse effect on the reproductive system. This pesticide is classified as an endocrine disruptor, meaning it is present in the environment or diet and interferes with normal hormone biosynthesis, signaling, or metabolism, thereby causing abnormal development, growth, or reproduction [3]. Endosulfan disrupts the function of androgen and estrogen receptors [14].

Sebastian and Raghavan showed that in mice, endosulfan exposure causes qualitative and quantitative defects during the course of spermatogenesis in a time-dependent manner. They observed increased levels of reactive oxygen species in the epididymis, which had an adverse effect on the integrity of sperm chromatin. Another consequence was a decrease in the number of spermatozoa in the epididymis and in the number of actively motile spermatozoa. All these phenomena led to infertility [81]. Exposure to endosulfan in male children may cause delayed puberty as well as disrupt the sex hormone balance. Saiyed et al. [16] studied 117 boys aged 10–19 years from a village in northern Kerala, India located in a valley below a hill with cashew plantations that had been aerially sprayed with endosulfan for over 20 years. The control group consisted of 90 boys not exposed to endosulfan. The mean serum endosulfan level was significantly higher in exposed children compared with controls (7.47 ppb vs. 1.37 ppb, respectively). Age-adjusted Tanner stage sexual maturity was negatively related to endosulfan exposure, and serum testosterone levels were lower while serum luteinizing hormone (LH) levels were higher in the study group than in the control group. Moreover, these authors found that congenital malformations of the genital tract (undescended testis, congenital hydrocele, and congenital inguinal hernia) were more common in the study group compared to the control group (5.1% vs. 1.1%, respectively), but the difference was not statistically significant.

Endosulfan may act as an estrogen-like compound at environmentally relevant doses [3]. Milesi et al. [82] showed that administration of low-dose endosulfan to neonatal female rats on days 1, 3, 5, and 7 after birth resulted in a reduced pregnancy rate and number of implantation sites after reaching sexual maturity. It cannot be ruled out that early exposure to endosulfan may also lead to reduced fertility in humans.

In the case of pregnant women, endosulfan may be transferred to the fetus and adversely affect intrauterine development, leading to reduced fetal growth. Moreover, endosulfan, by reducing progesterone levels, may contribute to preterm birth [18]. Pathak et al. [18] conducted a study in which they collected blood from mothers and umbilical cord blood immediately after delivery. They found significantly increased α-endosulfan content in cases of preterm delivery compared to full-term delivery (medians: in maternal blood 4.40 ng/mL vs. 2.38 ng/mL; in cord blood 3.03 ng/mL vs. 0.42 ng/mL, respectively). However, in contrast to the Pathak et al. study, Tyagi et al. [19] found no statistically significant differences in maternal serum endosulfan levels between preterm and full-term delivery (5.34 ppb vs. 5.76 ppb, respectively), nor were there any differences in endosulfan levels in placental tissues (11.30 ppb vs. 8.87 ppb, respectively). Therefore, it is uncertain whether endosulfan is associated with premature birth. Exposure of pregnant women to endosulfan may be associated with the development of neural tube defects (NTDs) in their children [83]. Ren et al. [17], in a study including 80 fetuses or newborns with NTDs and 50 healthy, non-malformed newborns, found an association between high levels of α-endosulfan in placentas and an increased risk of developing NTDs, anencephaly, and spina bifida. Kalra et al. [84] found that mothers giving birth to infants with NTDs had significantly higher blood endosulfan levels compared to control mothers, and furthermore, the median endosulfan levels in neonates with NTDs were higher than in their mothers. However, in their study, no significant associations between maternal and neonatal endosulfan blood levels were observed.

## 11. Endosulfan and Cancers in Humans

Endosulfan is associated with the risk of various cancers in humans [24,85]. There is a growing number of recent publications indicating an association between endosulfan and human cancers [21,22,24,28]. The mechanisms of the carcinogenic effect of endosulfan are rather poorly understood. The apoptotic effect of endosulfan on *HeLa* cancer cells and human hepatoma HepG2 cell lines was demonstrated in vitro, which was related to the generation of ROS [70].

Breast cancer is the most common malignancy in women. It is one of the leading causes of death worldwide. Endosulfan exposure has been associated with cases of this cancer [23]. Recently, Thammineni et al. [21] found significantly higher endosulfan content in breast cancer tissue compared to benign breast samples. In addition, the amount of endosulfan was higher in cases with lymph node metastases than in those without. Previously, Sharma et al. [22] detected increased plasma β-endosulfan levels in breast cancer patients compared to healthy individuals (0.80 ± 0.95 ng/mL vs. 0.03 ± 0.02 ng/mL, respectively). Furthermore, competitive binding affinity between β-endosulfan and the thyroxine binding site of transthyretin was revealed. This suggests the importance of competition between endosulfan and thyroxine, potentially leading to endocrine disruption associated with the development of breast cancer. Liu et al. [27] exposed the human breast adenocarcinoma MCF-7 cell line to different concentrations of endosulfan: the first group of cells to 1 μM, the second to 10 μM and the third to 20 μM. Exposure to endosulfan lasted 14 days. During this period, treatment solutions in different groups were refreshed every three days. They found that endosulfan activated the PI3K/AKT signaling pathway in MCF-7 cells in a dose-dependent manner, which promoted cell growth and induced EMT, thereby enhancing cell migration and invasion via the CCL5/CCR5 axis. Additionally, Kalinina et al. [86] found that 1 μM endosulfan reduced the protein levels of apoptosis regulators TP53INP1 (after 48 h of exposure) and APAF1 (after 24 and 48 h of exposure) in MCF-7 cells. Wang et al. [28] also reported that 12 h exposure to endosulfan (10 and 20 μM) promoted cell migration and invasion with the induction of epithelial–mesenchymal transition via the PTP4A3-dependent TGF-β signaling pathway in human prostate cancer cell lines PC3 and DU145.

Kiyani et al. [24] studied 89 randomly selected oncology patients from central Iran with different types of cancer (including breast, thyroid, prostate, skin, digestive system, uterus and ovaries, lung, liver and blood) and found that the presence of endosulfan in the blood increased the risk of death by 37%. These finding suggest that endosulfan exposure is associated with a worse prognosis in cancer patients.

## 12. Endosulfan and the Cardiovascular System

Endosulfan may lead to cardiovascular disease. Xu et al. [87] studied in vitro human umbilical vein endothelial cells (HUVECs) and showed that the permeability of HUVECs was increased after 48 h of endosulfan exposure in a dose-dependent manner when HUVECs were exposed to endosulfan at 20, 40, and 60 μM doses. This compound enhances endothelial cell permeability through both transcellular and paracellular pathways. This causes the loss of endothelial barrier function. Additionally, Wei et al. [72] found that endosulfan at concentrations of 2, 6, 12, 16, and 32 μg/m reduced cell viability in HUVECs after 24 h of exposure in a dose-dependent manner. Endosulfan maintained these cells in the S and G2/M phases and also inhibited HUVECs’ proliferation. It has been shown in rats that oral endosulfan administration at a dose of 5 or 10 mg/kg for 21 days can cause blood hypercoagulation by initiating the extrinsic coagulation pathway, resulting from endothelial cell damage. It promotes the conversion of fibrinogen to fibrin, increases platelet aggregation, and also reduces the activity of antithrombin III [88].

## 13. Other Endosulfan Effects on Human Health

Endosulfan can cause rhabdomyolysis, which then leads to renal tubular damage, resulting in acute tubular necrosis and ultimately renal failure [20]. In vitro in human renal tubular epithelial cells HK-2, endosulfan induced apoptosis by regulating the expression of caspase-3, BAX, and APAF-1; additionally, mitochondrial cytochrome c was released into the cytosol. Endosulfan induced an inflammatory response, showing an increase in the secretion and expression levels of IL-6/IL-8 mRNA [89].

Endosulfan has an adverse effect on the functioning of the pituitary gland. It affects the voltage-dependent L-type Ca^2+^ channels. Caride et al. [90] showed in pubertal male rats that oral administration of endosulfan caused a decrease in the gene expression of thyroid-stimulating hormone (TSH), growth hormone (GH), prolactin, and luteinizing hormone (LH). Additionally, endosulfan increased the expression of nitric oxide synthase 1 and 2 at the pituitary, suggesting that nitrosative stress may be related to endosulfan toxicity in the pituitary. Coskun et al. [15] reported adrenocorticotropic hormone (ACTH) deficiency in one patient exposed to endosulfan 3 months after acute poisoning and GH deficiency in another patient.

Wang et al. [91] showed that in healthy women, the intake of β-endosulfan as well as total endosulfan from contaminated plant foods was positively correlated with serum IL-8 levels. IL-8 is a proinflammatory cytokine, and its levels are increased in patients with various autoimmune diseases and cancers.

## 14. Endosulfan Acute Poisoning and Its Treatment

Cases of acute poisoning described in the literature were most often caused by oral exposure to endosulfan (as a plant protection product or with contaminated food). Frequently, acute poisoning was caused by a suicide attempt [25,52,92].

Typical symptoms of endosulfan poisoning include nausea, vomiting, headache, dizziness, paresthesias, focal myoclonic seizures, and generalized seizures [1,25,26]. Moon and Chun [25] reported 52 patients hospitalized with acute poisoning after oral ingestion of endosulfan. The most common symptom was generalized tonic–clonic seizures (92.3% of patients). They began within 20 to 210 minutes of ingestion and lasted 1 to 7 days. Most complications occurred within 48 h of ingestion: rhabdomyolysis (59.6%), hepatic toxicity (36.5%), hypotension (34.6%), acute kidney injury (26.9%), and thrombocytopenia (21.2%). These complications lasted for a variable length of time and completely resolved in the surviving patients. Liver function tests returned to normal within 18 days of ingestion in the surviving group, while patients in the non-surviving group continued to have ALT levels of more than 100 U/L until death. Hypotension, on the other hand, persisted for 8 days and was refractory to treatment until death in the non-survivors. Mortality in the study group was 30.7%. The causes of death were the following: refractory status epilepticus (twelve patients), multiple organ failure (two patients), and acute renal failure with metabolic acidosis (two patients). Death from status epilepticus occurred within 3 h to 3 days after ingestion, and from multiple organ failure or acute renal failure within 3 to 8 days after ingestion. The literature also describes a fatal case of a 26-year-old female patient who, following endosulfan poisoning, developed status epilepticus, followed by intravascular hemolysis and disseminated intravascular coagulation (DIC) [92].

Endosulfan poisoning can cause malignant hyperthermia. Jain et al. [93] described the case of a man who took endosulfan orally with the intention of committing suicide. He had a nasopharyngeal temperature of 40 °C 8 h after taking the poison. Malignant hyperthermia was confirmed by caffeine halothane contraction test. Normally, after muscle cell contraction, Ca^2+^ ions are rapidly sequestered into the sarcoplasmic reticulum. Endosulfan, however, by inhibiting the action of Ca- and Mg-ATPases, prevents this uptake of Ca^2+^ ions. The accumulation of Ca^2+^ leads to excessive muscle contraction and hyperthermia.

Interestingly, there is a case report of a 2-year-old girl whose mother applied endosulfan to the scalp to treat lice. After 2 h, the child experienced continuous generalized tonic–clonic seizures, which lasted for 1 h, and they were stopped by intravenous administration of diazepam [54].

There is no known antidote to endosulfan. Care of poisoned patients is symptomatic treatment. Management is based on symptomatic treatment. In patients who took high doses of endosulfan, prophylactic anticonvulsant therapy is essential. Initial treatment for acute endosulfan poisoning includes gastric lavage and activated charcoal administration [94,95]. However, Moses and Peter [95] recommended that gastric lavage and activated charcoal administration should probably be restricted to patients presenting shortly after poisoning (< 1 h). The most common cause of death after acute endosulfan poisoning is refractory status epilepticus. Therefore, priority should be given to treating seizures. Moon and Chun [25] suggested prophylactic administration of anticonvulsants in patients who have ingested more than 35 g of endosulfan. There are no studies on the optimal anticonvulsant treatment algorithm for endosulfan-induced seizures. Benzodiazepines [54,96] and barbiturates seem to be useful as first- and second-line drugs [54]. They are agonists of GABA-A receptors, which facilitate the influx of chloride ions and consequently increase the inhibition of GABA, which was disrupted by endosulfan [54]. However, in the Moon and Chun [25] study, benzodiazepine with a total dose of 30–60 mg effectively controlled seizures in only 8.3% of patients with seizures, while 91.7% of patients with seizures required more than two anticonvulsants.

During further treatment, the patient’s clinical condition should be carefully monitored, and both procedures and medications used depend on the clinical course. Most patients recover after symptomatic treatment, but unfortunately, fatal cases also occur. Therefore, it is important to provide appropriate care for patients and identify those at particular risk [56]. Moon et al. [56] in a study of patients hospitalized due to oral endosulfan ingestion, found that independent factors predicting death during hospitalization were an ingested endosulfan dose exceeding 35 g and low arterial blood pH. Respiratory failure due to status epilepticus is a common cause of death, and its treatment may require intubation and mechanical ventilation. Another serious problem may be metabolic acidosis, which requires appropriate treatment, including the need for hemodialysis in some patients [76,94]. Boereboom et al. [97] presented a case report in which they described the failure of hemoperfusion in a 43-year-old male patient poisoned with endosulfan.

## 15. Towards Safer Alternatives to Endosulfan

In light of the environmental persistence and toxicity associated with endosulfan, there has been a significant shift towards safer pest management strategies. Biopesticides, derived from natural sources such as plants, microorganisms, and minerals, offer targeted pest control with reduced environmental impact. For instance, neem-based products containing azadirachtin have demonstrated efficacy against a variety of insect pests while exhibiting low toxicity to non-target organisms [98]. Similarly, pyrethrins extracted from Chrysanthemum flowers are effective insecticides that degrade rapidly in the environment, minimizing ecological risks [99,100,101].

Beyond biopesticides, agroecological practices emphasize ecosystem-based approaches to pest management. These methods, endorsed by the Stockholm Convention, focus on enhancing biodiversity and natural pest control mechanisms, reducing reliance on chemical inputs. Such strategies not only mitigate the adverse effects associated with synthetic pesticides but also promote sustainable agricultural systems [99,102,103,104].

## 16. Conclusions

Endosulfan’s physicochemical properties contribute to its persistence in the environment and its potential for bioaccumulation. Understanding these characteristics is crucial for assessing its environmental impact and potential health risks.

Due to the harmful, serious impact of endosulfan on human health, it is necessary to monitor the presence of pesticide residues such as endosulfan in the environment and especially in food. Particular attention should be paid to the exposure of children who are more susceptible, among others, due to not fully developed organs and lower detoxification capacity.

Coordinated international and regional efforts are needed as part of a comprehensive approach to mitigating the risks of endosulfan, aimed at protecting both the environment and human health.

Understanding the scope of health impacts and ongoing risks is crucial for policymakers, researchers, and public health authorities aiming to protect people from legacy pollutants. Extensive knowledge of endosulfan’s long-term impacts is essential for effective health risk assessment, environmental monitoring, and the promotion of safer alternatives.

## Data Availability

The data are contained within the article.

## Abbreviations

The following abbreviations are used in this manuscript:

AChE	Acetylcholinesterase
ACTH	Adrenocorticotropic hormone
ALS	Amyotrophic lateral sclerosis
ALT	Alanine aminotransferase
AST	Aspartate aminotransferase
BMI	Body mass index
CAT	Catalase
CNS	Central nervous system
COP5	Fifth meeting of the Conference of the Parties
DIC	Disseminated intravascular coagulation
EPA	Environmental Protection Agency
EU	European Union
GABA	γ-aminobutyric acid
GH	Growth hormone
GSH	Glutathione
GST	Glutathione-S-transferase
HUVEC	Human umbilical vein endothelial cells
IL	Interleukin
LC_50_	Lethal concentration 50%
LD50	Median lethal dose
LH	Luteinizing hormone
MN	Micronuclei
NOEL	No Observed Effect Level
NTD	Neural tube defects
PBMC	Peripheral blood mononuclear cells
POPs	Stockholm Convention on Persistent Organic Pollutants
ROS	Reactive oxygen species
SCE	Sister chromatid exchanges
SCG	Single-cell gel electrophoresis
SOD	Superoxide dismutase
TSH	Thyroid-stimulating hormone
WHO	World Health Organization
